# Dynamic Succession of Natural Microbes during the Ecolly Grape Growth under Extremely Simplified Eco-Cultivation

**DOI:** 10.3390/foods13101580

**Published:** 2024-05-18

**Authors:** Yinting Ding, Lin Wang, Hua Wang, Hua Li

**Affiliations:** 1 College of Enology, Northwest A&F University, Xianyang 712100, China; dyting@nwafu.edu.cn (Y.D.); maggie_wongkaixin@163.com (L.W.); wanghua@nwafu.edu.cn (H.W.); 2 China Wine Industry Technology Institute, Yinchuan 750021, China; 3 Shaanxi Engineering Research Center for Viti-Viniculture, Xianyang 712100, China; 4 Engineering Research Center for Viti-Viniculture, National Forestry and Grassland Administration, Xianyang 712100, China

**Keywords:** extremely simplified eco-cultivation, microbial diversity, microbial succession, continuum, sustainable development

## Abstract

The composition and continuous succession of natural microbial communities during grape growth play important roles in grape health and flavor quality as well as in characterizing the regional wine terroir. This study explored the diversity and dynamics of fruit epidermal microbes at each growth and developmental stage of Ecolly grapes under an extremely simplified eco-cultivation model, analyzed microbial interactions and associations of weather parameters to specific communities, and emphasized metabolic functional characteristics of microecology. The results indicated that the natural microbial community changed significantly during the grape growth phase. The dominant fungal genera mainly included *Gibberella*, *Alternaria*, *Filobasidium*, *Naganishia*, *Ascochyta*, *Apiotrichum*, *Comoclathris,* and *Aureobasidium*, and the dominant bacterial genera mainly contained *Sediminibacterium*, *Ralstonia*, *Pantoea*, *Bradyrhizobium*, *Brevundimonas*, *Mesorhizobium*, *Planococcus,* and *Planomicrobium*. In summary, filamentous fungi gradually shifted to basidiomycetous yeasts along with fruit ripening, with a decline in the number of Gram-negative bacteria and a relative increase in Gram-positive bacteria. The community assembly process reflects the fact that microbial ecology may be influenced by a variety of factors, but the fungal community was more stable, and the bacterial community fluctuated more from year to year, which may reflect their response to weather conditions over the years. Overall, our study helps to comprehensively profile the ecological characteristics of the grape microbial system, highlights the natural ecological viticulture concept, and promotes the sustainable development of the grape and wine industry.

## 1. Introduction

The origin and domestication of grapes can be traced back to tens of thousands of years ago. With the development of human activities and agricultural civilization, grapes gradually spread all over the world and became an economically important fruit that is widely planted worldwide [1,2]. The determination of appropriate varieties and cultivation techniques to ensure the grape quality and productivity according to the climatic types of different ecological zones has contributed to the development of viticulture [3,4]. The growth and ripening of grapes naturally drive the vinification techniques, thus contributing to the development of the entire grape and wine industry. Therefore, the transformation of grapes into wine is a natural, unified continuum, with the quality of the grape berries being the basis for determining the wine flavor quality [5,6].

The complex and diverse microbial community naturally exists in the environment in which grapes are grown, especially the microbiome that attaches to the grape epidermis. Theoretically, when the berries fall to the ground and split open after ripening, the sugars and nutrients flow out, in which the yeasts on the grape surface become active and the winemaking process begins, with no human intervention at all. Hence, it can be stated that wine is a human discovery rather than a human invention. Grapes and their microbiota have co-evolved over long periods of cultivation and reproduction as well as in response to various selection pressures [7,8]. These microbial communities perform important roles in grapevine development, fruit health, resistance and adaptation to environmental stresses, as well as induction of plant defense mechanisms [9,10].

The community composition of the grape microbial ecosystem has been extensively studied, with the main results focusing on filamentous fungi, yeasts, and bacteria, which colonize the exterior and interior of the grape organ, and can be categorized into beneficial flora, disease-causing pathogens, and drifting species [11,12,13]. Studies have revealed that beneficial microorganisms promote the utilization of mineral nutrients in grapes, synthesize hormones in response to environmental stresses, antagonize pathogenic microorganisms for biocontrol through predation and competition, and even stimulate defense mechanisms by inhibiting and degrading virulent components of pathogens [14,15]. For example, *Fusarium delphinoides*, *Trichoderma harzianum*, and *Bacillus subtilis* have been proven to be capable of enzymatic activity to produce extracellular lytic enzymes or antimicrobial peptides for efficient control of the downy mildew pathogen (*Plasmopara viticola*). However, grapes are highly susceptible to a variety of diseases and pests during growth and ripening, reducing quality and production and causing significant economic losses. In addition, in their natural state, a large number of grape microbes are neutral drifting species that do not seriously jeopardize grape health but instead have a diluting effect on the population of plant pathogens by increasing microbial diversity. There is a growing view that under favorable ecological conditions, the grape natural microbial ecosystem can remain relatively stable, and the dynamic and balanced interactions between microbes and between grapes and microbes can ensure growth health and material accumulation [16,17]. It has also been found that the presence and metabolic activity of certain organisms on the grape epidermis may antagonize other species, while bioactive molecules secreted by endophytic and phyllosphere yeasts can also replace the action of essential agrochemicals [18]. Moreover, natural yeasts may have biocontrol activity against filamentous fungi.

The negative impacts of the heavy use of fungicides, insecticides, and fertilizers in traditional viticultural systems on the grape and wine industries have been increasingly appreciated [19]. The addition of these chemicals results in a monolithic and fragile grape ecosystem, damages the natural defense network of the vineyard, reduces the microbial community diversity, leads to an increase in pathogen resistance over the years, and inhibits the production of the secondary metabolites of the grapes themselves, which can even obscure the wine origin characters and lead to a homogenization of the wine flavor. Extensive pesticide residues will affect the quality of grapes and wine and may harm consumer health. It has been found that the grape natural microbes, especially non-*Saccharomyces*, are able to express non-random dispersion patterns of different origin sources, conferring microbial terroir characteristics to the wine, which are perceived in the flavor metabolite composition and sensory properties [20,21]. Therefore, healthy and ripe grape berries harvested at the right time will have all the conditions to transform themselves into wine.

With climate change becoming a growing problem, traditional viticulture will face climatic, economic, environmental, social, and food security challenges, which require the exploration of a high-quality, sustainable industrial model [22,23]. Moreover, more consumers are paying more attention to the concept of nature and health, so organic, biodynamic, and natural wines continue to emerge and become increasingly popular and appreciated by consumers. However, biodynamic and organic concepts still involve the addition of exogenous substances, such as copper preparations and compost preparations [24,25]. Taking into account the current cultivation situation in Chinese wine regions, we propose an extremely simplified eco-cultivation model, whose core elements include grapevine form control, natural grass cover, branch cover in intra-row, and wintering with branch on trellis. The model works by minimizing unnecessary treatments (pesticides, fertilizers, heavy irrigation, etc.), thus improving the microclimate of the vineyards, preserving biodiversity, improving ecological function and landscape value, and guaranteeing optimal growing conditions for the grapes [4,26].

This study is dedicated to exploring the species composition, dynamic succession, and potential metabolic functions of the natural microbial community of Ecolly (*Vitis vinifera* L.) grapes during fruit development under an extremely simplified eco-cultivation model. It will provide a new perspective for understanding and generalizing the succession patterns of microbial communities and their impact on grape health, optimizing viticulture management practices, characterizing the wine microbial terroir, and thereby achieving sustainable, high-quality development of the grape and wine industries.

## 2. Materials and Methods

### 2.1. Microbiological Sampling

The Ecolly (*Vitis vinifera* L.) grapes used in this experiment were collected in 2021 from the vineyard of Sunshine Tianyu Winery (39°38′ N, 106°76′ E), Wuhai, Inner Mongolia, China. The vineyard is located in a temperate continental monsoon climate with hot, dry summers, high temperatures, and little precipitation, which are generally sufficient for healthy and fully ripened grapes that are less susceptible to microbial diseases. The experimental vineyards in this study were planted under an extremely simplified cultivation model, where fungicides, insecticides, and chemical fertilizers were usually not used during the growing season to maintain the natural balance of the grape microbial ecosystem.

In this study, microbial samples were collected from the grape epidermis at fruit-set (A, E-L stage 27), veraison-early (B, E-L stage 33), veraison-end (C, E-L stage 35), mid-maturity (D, E-L stage 37), and harvest (E, E-L stage 38) in a modified Eichhorn–Lorenz (EL) system to investigate the composition and dynamics of the grape microbiome (Supplementary Figure S1). For each of the above growth stages, we collected microbes from the berry epidermis using sterile cotton swabs dipped in saline, sampling three biological replicates per stage, with each replicate collected from five sampling sites to be spread throughout the vineyard, with three randomly selected vines at each sampling point. The sampling was carried out when 80% of the clusters reached the stages mentioned above and was collected in combination with the rightward and backward sides of the grapevine and the upper, center, and lower parts of the clusters. As a result, a total of 15 microbial samples were obtained, which were placed in sterilized centrifuge tubes and then rapidly transported to the laboratory using a refrigerated box filled with dry ice and stored at −80 °C before DNA extraction was performed.

### 2.2. DNA Extraction and Processing

The total DNA of the grape epidermal microbiome was extracted according to the instructions of the HiPure Soil DNA Kits. The purity and integrity of the DNA were checked by 2% agarose gel electrophoresis and NanoDrop 2000 microspectrophotometer. The DNA was extracted and stored at −20 °C until further analysis. The V3–V4 region of the bacterial 16 rRNA gene was amplified using the specific primer pair 341F(5′-CCTACGGGNGCWGCAG-3′)/806R(5′-GGACTACHVGTGGGATCTAAT-3′) with barcode. The ITS2 region of fungal internal transcribed spacer (ITS) was amplified using the primer pair ITS3_KYO2(5′-GATGAGAGYACAGYRAA-3′)/ITS4(5′-TCCTGCTTATATGATATGC-3′). The PCR reaction system consisted of 5 µL of 10× Buffer KOD, 5 µL of 2 mM dNTPs, 3 µL of 25 mM MgSO_4_, 1.5 µL of each of the forward and reverse primers, 100 ng of template DNA, and make-up water to 50 µL. The amplification was performed under the following conditions: 94 °C for 2 min, then 30 cycles of 10 s at 98 °C, 30 s at 62–66 °C, and 30 s at 68 °C with a final extension of 5 min at 68 °C. Amplicons were extracted from 2% agarose gels, purified using AMPure XP Beads (Beckman Agencourt, Brea, CA, USA) under the manufacturer’s instructions, and quantified using the ABI StepOnePlus Real-Time PCR System (Life Technologies, Foster City, CA, USA). According to the standard protocols, purified amplicons were pooled in an equimolar and paired-end sequenced (PE250) using Illumina Novaseq 6000 platform.

### 2.3. Bioinformatics Analysis

Raw reads that contained low-quality reads with more than 10% of unknown nucleotides or with 50% of low-quality (Q-value ≤ 20) bases were filtered by fastp (version 0.18.0). Paired-end clean reads were merged using FLSAH (version 1.2.11), and noisy sequences of raw tags were filtered by QIIME (version 1.9.1). All chimeric tags were removed using UCHIME algorithm (version 4.2), and using the UPARSE (version 9.2.64) pipeline, the clean tags were clustered into operational taxonomic units (OTUs) with an average similarity of 97%. A naive Bayesian model with a confidence threshold value of 0.8 was used to classify the representative OTU sequences into organisms using RDP classifier (version 2.2) based on the SILVA database (version 132) or the UNITE database (version 8.0). Regarding the DNA sequencing data, we uploaded them to the SRA database at NCBI under accession numbers PRJNA1046263 (fungal ITS sequences) and PRJNA1046273 (bacterial 16S rRNA gene sequences).

### 2.4. Statistical Analysis

Microbial community alpha diversity was calculated using QIIME (version 1.9.1), and Tukey’s HSD in R project Vegan package (version 2.5.3) was used to compare between groups. The principal coordinate analysis (PCoA) of weighted UniFrac and Bray–Curtis distances was generated in the R project Vegan package (version 2.5.3) and plotted in the R project ggplot2 package (version 2.2.1). Permutational multivariate analysis of variance (PERMANOVA) and Procrustes test were calculated in the R project Vegan package (version 2.5.3).

Community composition was visualized by circos plot and pie plot in R project circlize package (version 0.69-3) and ggplot2 package (version 2.2.1). Significant taxonomic differences in microbial genera during grape growing stages were tested using linear discriminant analysis (LDA) and effect size (LEfSe) (LDA > 3). The original table of OTUs was filtered to include only OTUs with relative abundance higher than 0.01% to reduce LEfSe complexity. A null model based on beta nearest classification index (βNTI) with 999 iterations and Raup–Crick index based on Bray–Curtis was used to study the assembly process of microbial communities. Briefly, |βNTI| ≥ 2 is defined as the dominant deterministic process, and |βNTI| < 2 dominates the stochastic process. The deterministic and stochastic processes were categorized into five ecological processes based on βNTI and the Raup–Crick index (RC Bray), including heterogeneous selection (βNTI < −2), variable selection (βNTI > 2), dispersal limitation (|βNTI|< 2 and RC Bray > 0.95), homogeneous dispersal (|βNTI|< 2 and RC Bray < −0.95), and undominated (|βNTI|< 2 and |RC Bray |< 0.95) [27,28].

The following weather parameters for different growth stages were obtained through the weather monitoring system of the vineyard: EnV1, mean temperature (°C); EnV2, mean high temperature (°C); EnV3, mean low temperature (°C); EnV4, precipitation (mm); EnV5, relative moisture (%); EnV6, evaporation (mm); EnV7, solar radiation (J/m2); and EnV8, sunlight hours (h). Redundancy analysis (RDA) and Mantel test were used to analyze the effects of weather parameters on microbial communities. Variance partition analysis (VPA) was drawn through the R project Vegan package (version 2.5.3) to analyze the explanation degree of the total variation in species distribution by environmental factors. Co-occurrence networks between microbial genera and between microbes and weather conditions were constructed using Gephi (version 0.9.7) based on Spearman correlation coefficients. Metabolic functional analysis of fungi and bacteria was predicted using PICRUSt2 from MetaCyc pathway database and Integrated Microbial Genomes database (IMG), respectively, and abundance was exhibited by heat map using the pheatmap package in R project.

## 3. Results

### 3.1. Grape Developmental Stage Significantly Influences Microbial Diversity

Through data quality control, 1,934,527 and 1,726,796 effective fungal tags and bacterial tags were obtained during the growth and development of grape berries, respectively, with an average of 128,969 effective fungal tags and 115,120 bacterial tags present in each sample. The comparison of the databases clustered 1169 fungal OTUs and 5600 bacterial OTUs with 97% similarity. The rarefaction curves of the sequenced samples all leveled off, and the coverage of high-quality sequences was higher than 99%, indicating that the quantity of sequencing data was sufficient and the depth of sequencing can satisfy the analysis of microbial community diversity and richness (Supplementary Figure S2).

A comprehensive assessment of successive changes in epiphytic microbial community richness and diversity during the grape growing season is presented in Supplementary Table S1. It was found that both the diversity and richness of the fungal community differed significantly during the grape growth period by analyzing the changes in Shannon and Chao1 indices. For fungi, Shannon diversity of fungal communities was significantly higher at the fruit-set than at other stages, but the Chao1 index remained low, suggesting that fungal species at this time were relatively fewer, but the various types of fungi remained even in abundance. Fungal diversity decreased significantly before and after the color change period, but species richness remained stable, suggesting that changes in the physiological state of the fruit may have influenced the colonization state of specific fungal flora, thereby selectively increasing the fungal taxa associated with the onset of grape ripening. The richness and diversity of the fungal community increased significantly at mid-maturity and harvest, accompanied by the softening of the ripening grape berries and the increased availability of nutrients. Relative to the bacterial community, we found that the bacterial community was richer and more diverse at the veraison stage, while the diversity of the bacterial community exhibited a significant decrease from the mid-maturity to the harvest stages. Overall, the balance of the microbial community in the grape epidermis was significantly altered during growing stages (Supplementary Figure S3: F value = 7.371, *p* = 0.005), with a significant imbalance in species evenness especially at the veraison-early stage (the smaller the ratio, the greater the degree of evenness), but thereafter, the microbial community gradually came into balance.

**Figure 1:**
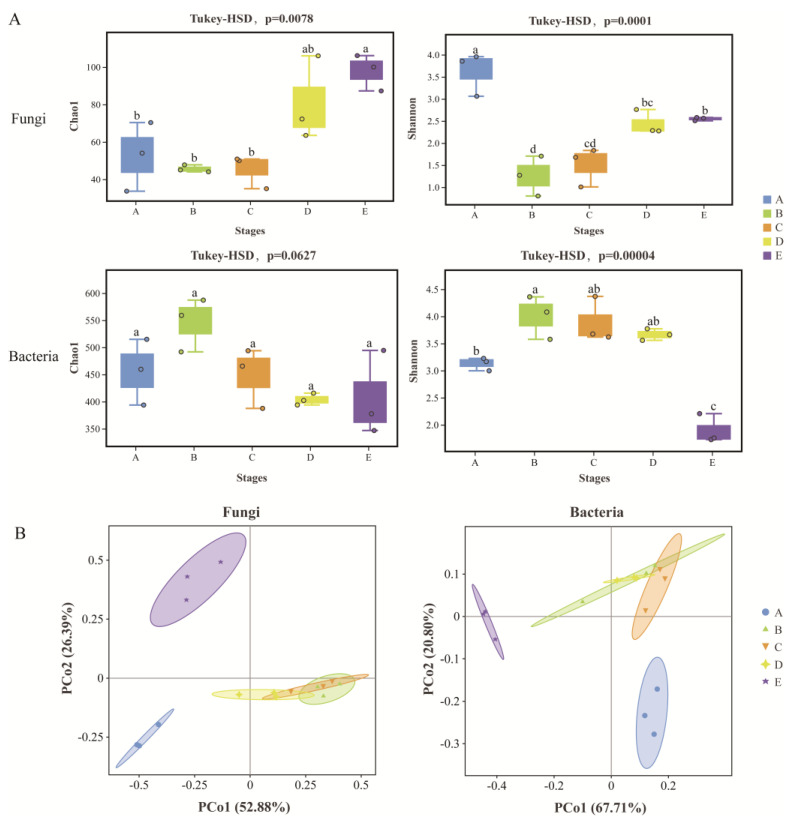
Changes in the diversity of microbial communities during the growth and development of grape berries. (**A**) Chao1 and Shannon indices changes in fungi and bacteria; (**B**) principal coordinate analysis (PCoA) based on OTU level; fungi: Bray–Curtis; and bacteria: weighted UniFrac. Note: Different lowercase letters “a–d” indicate significant differences (Tukey-HSD, *p* < 0.05).

The differences in microbial community structure during fruit development were assessed by PCoA based on OTU levels. The results indicated that the total explanations against the fungal and bacterial community structure reached 79.27% and 88.51%, respectively, where it was observed that the fungal and bacterial communities at the fruit-set and harvest stages were clearly separated from the rest of the period, while samples of fungi and bacteria at the veraison stage as well as at the mid-maturity stage were clustered together and showed similar microbial community characteristics (Figure 1B). Statistical results revealed that the community structure of grape epiphytic fungi and bacteria was significantly affected by the development stage (Table 1: PERMANOVA; fungal Bray–Curtis, R^2^ = 0.7943, and *p* = 0.001; bacterial weighted UniFrac, R^2^ = 0.8807, and *p* = 0.001). Meanwhile, Procrustes test analysis identified potential correlation and consistency between fungal and bacterial community structure in response to grape developmental stages (Supplementary Figure S4; Bray–Curtis, M^2^ = 0.2551, R = 0.863, *p* = 0.001).

### 3.2. Microbial Community Composition and Succession

**Figure 2:**
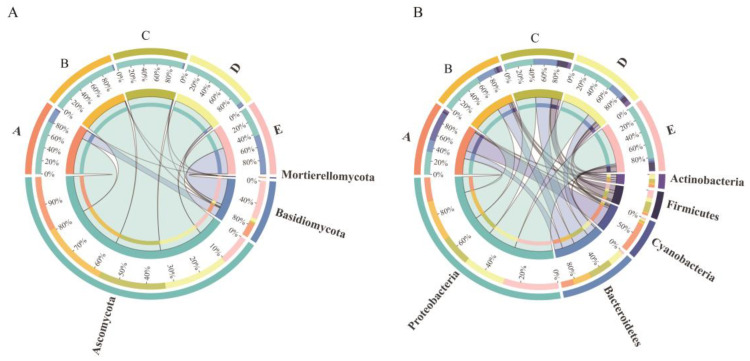
The relative abundance of microbial dominant phylum during grape growth: (**A**) fungi and (**B**) bacteria. A: Fruit-set; B: veraison-early; C: veraison-end; D: mid-maturity; and E: harvest.

In this study, a total of 5 fungal phyla and 21 bacterial phyla, categorized into 117 fungal genera and 490 bacterial genera, were identified in the fruit epidermis of grapes during the growing season. *Ascomycota* and *Basidiomycota* are the main fungal phyla present in the grape epidermis. During berry growth, the proportion of Ascomycota decreased, and the abundance of *Basidiomycota* increased. In particular, the abundance of Ascomycota was absolutely dominant before the harvest, with relative abundance of 78.75–98.70%, but at harvest stage, *Basidiomycota* became the dominant fungal taxa, with a relative abundance of 56.49%, whereas the relative abundance of *Ascomycota* decreased to 43.50% (Figure 2A). For the bacterial taxa, the dominant bacterial phyla were *Proteobacteria*, *Bacteroidetes*, and *Cyanobacteria*, whose average relative abundance was 56.74%, 20.82%, and 10.74%, respectively. *Cyanobacteria* and *Proteobacteria* were predominant at the fruit-set stage, after which the number of *Cyanobacteria* decreased rapidly and *Proteobacteria* became the obvious dominant bacterial group, with the relative abundance of *Proteobacteria* reaching 82.03% at the harvest stage (Figure 2B).

Throughout the grape growth and development stage, fungal genera with high mean relative abundance mainly included *Gibberella* (43.32%), *Alternaria* (12.02%), *Filobasidium* (8.83%), *Naganishia* (4.11%), *Ascochyta* (2.65%), *Apiotrichum* (2.37%), *Comoclathris* (1.47%), *Aurcobasidium* (0.99%), *Saccharomyces* (0.81%), and *Malassezia* (0.70%), and bacterial genera included *Sediminibacterium* (19.92%), *Ralstonia* (18.44%), *Pantoea* (17.67%), *Bradyrhizobium* (5.87%), *Brevundimonas* (2.52%), *Mesorhizobium* (2.04%), *Planococcus* (1.72%), *Planomicrobium* (1.44%), *Bacillus* (1.06%), and *Enterococcus* (1.00%).

**Figure 3:**
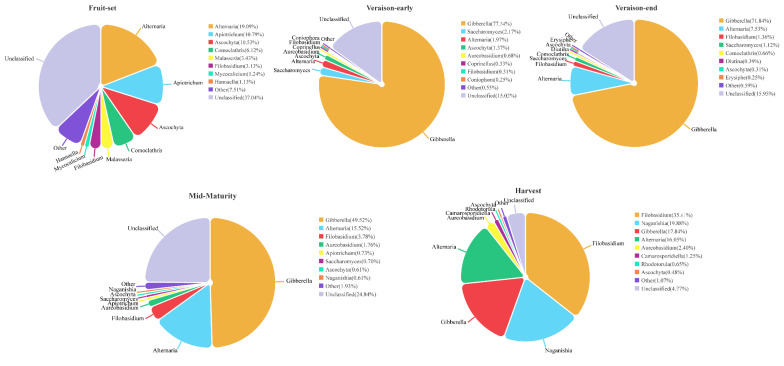
The relative abundance of dominant fungal genera at each stage of grape berry growth.

**Figure 4:**
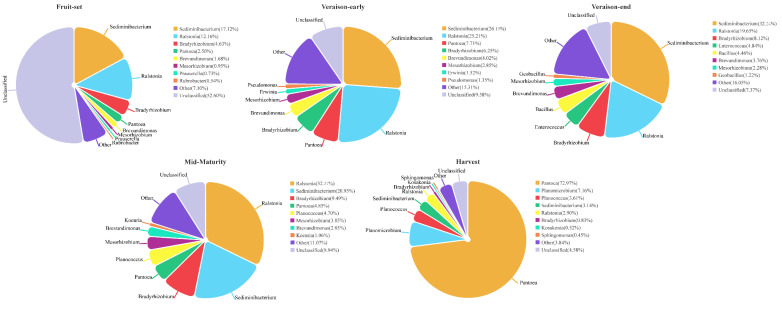
The relative abundance of dominant bacterial genera at each stage of grape berry growth.

The dominant fungal and bacterial genera at each grape development stage are shown in Figure 3 and Figure 4, reflecting the succession of natural microbes accompanying fruit growth. Among them, the dominant fungal genera at the fruit-set stage were more evenly distributed, mainly characterized by the filamentous fungi *Alternaria*, *Apiotrichum*, *Comoclathris,* and *Ascochyta*. At this time, these fungi do not significantly affect grape health, but their presence provides conditions for successive fungal succession. The veraison-early stage was recognized by significant changes in grape physiology, which led to an increase in the relative abundance of specific fungi, such as *Gibberella*, whose relative abundance jumped to 77.34% with absolute dominance, and also the presence of small amounts of *Saccharomyces*, *Alternaria*, *Ascochyta,* and *Aureobasidium*. Thereafter, *Gibberella* remained numerous at the veraison-end stage with a slight increase in the abundance of *Alternaria* and *Filobasidium*, and a trend towards a decrease in *Saccharomyces*. At mid-maturity, the relative abundance of *Gibberella* decreased and the numbers of *Filobasidium*, *Aureobasidium*, and *Naganishia* continued to increase. At harvest stage, the relative abundance of *Filobasidium* and *Naganishia* increased substantially, while the relative abundance of *Gibberella* decreased to 17.84%, the number of *Aureobasidium* increased slightly, and *Alternaria* remained relatively stable. Overall, the number of *Gibberella* gradually decreased and *Alternaria* steadily increased with fruit ripening. Moreover, the populations of *Filobasidium*, *Aureobasidium*, and *Naganishia* gradually dominated, and the increase in the populations of these beneficial grape bacteria inhibited the spread of fungi such as *Alternaria*, *Ascochyta*, and *Erysiphe*, so that harvesting at the right time ensured the health and quality of the grape berries (Figure 3).

The relative abundance of bacterial genera was more evenly distributed between the fruit-set stage and veraison-early stage, dominated by Gram-negative bacteria such as *Sediminibacterium*, *Ralstonia*, *Bradyrhizobium*, *Pantoea,* and *Brevundimonas*, which showed a tendency to increase in their relative abundance. At the veraison-end stage, the relative abundance of *Sediminibacterium* and *Ralstonia* remained dominant, in addition to a certain number of Gram-positive bacteria such as *Enterococcus* and *Bacillus*. *Ralstonia* became the most abundant bacterial genus at mid-maturity, with the combined relative abundance of *Sediminibacterium* and *Ralstonia* reaching a peak (more than 52%), *Bradyrhizobium* remaining relatively stable, and small amounts of *Pantoea* and *Planococcus* still present. At the harvest stage, the relative abundance of *Pantoea* jumped to 72.97%, becoming the absolute dominant bacterium, *Sediminibacterium*, *Ralstonia*, and *Bradyrhizobium* were drastically less in number, and Gram-positive bacteria such as *Planococcus* and *Planomicrobium* were present in higher numbers at the ripening stage. In general, the abundance of Gram-negative bacteria was higher when the grapes were unripe. As the fruit ripened and the environmental conditions of the grape epidermis changed, Gram-negative bacteria decreased in number, and Gram-positive bacteria increased in abundance, and they may have a role in the accumulation of fruit matter and the fermentation process (Figure 4).

**Figure 5:**
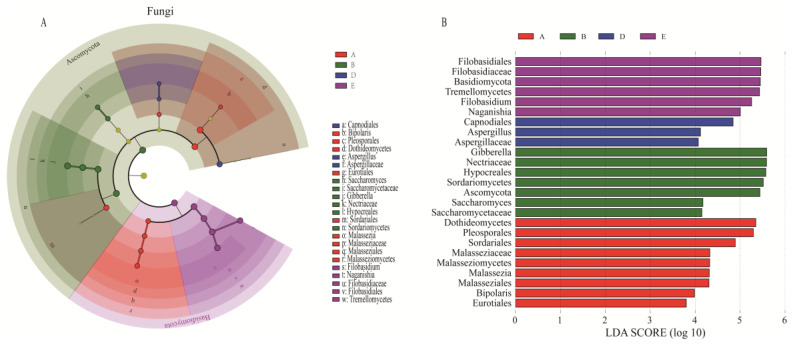
Linear discriminant analysis (LDA) and effect size (LEfSe) to determine fungal markers for each growth stage. (**A**) Evolutionary branching maps characterize the biological taxonomic hierarchy of significantly different fungal taxa; and (**B**) linear discriminant analysis (LDA) demonstrates the effect size of differential fungal genera. A: Fruit-set; B: veraison-early; C: veraison-end; D: mid-maturity; and E: harvest.

The LEfSe of indicator fungi and bacteria for the presence of significant differences in the various grape growth stages was carried out (Figure 5 and Supplementary Figure S5). The fungal genera during the growing season showed *Malassezia* and *Bipolaris* as the characteristic fungal genera during the fruit-set stage; the veraison-early was mainly enriched with *Saccharomyces* and *Gibberella*; *Aspergillus* was the indicator fungus during the mid-maturity stage; and the biomarker fungi during the harvest stage were *Naganishia* and *Filobasidium*. Similarly, the indicator bacterial genera for the fruit-set stage included *Tatumella*, *Exiguobacterium*, *Gluconobacter*, and *Cutibacterium*; and the characteristic bacteria for the veraison-early stage consisted of a total of 11 genera, mainly *Brevundimonas*, *Acinetobacter*, *Bosea*, and *Sphingomonas*, indicating again that the microbial community changed significantly during the veraison-early stage. *Paenibacillus*, *Modestobacter*, *Chryseobacterium*, and *Bacillus* were predominantly enriched at the veraison-end stage; the biomarker bacteria at mid-maturity consisted of eight genera, mainly *Ralstonia*, *Mesorhizobium*, *Planococcus*, and *Bradyrhizobium;* while the characteristic bacterial genera at the harvest stage were *Pantoea*, *Kosakonia*, and *Marinilactibacillus*. Above all, the indicator microbes present at different times are common and important microbial genera, and their succession pattern reflects the microbial characteristics of the grapes during a specific growth phase. The fact that they are not only highly persistent but also drive the seasonal alternation of microbial communities along with grape ripening will be important for us to control and utilize natural microorganisms to safeguard grape health.

### 3.3. Microbial Community Assembly Processes

Microbial assembly processes during fruit growth were investigated by a null model based on βNTI and Raup–Crick indices (Supplementary Figure S6). The results suggested that both fungal and bacterial communities on the fruit surface were mainly dominated by stochastic processes, especially undominated processes (−2 < βNTI < 2, −0.95 < RC Bray < 0.95). It indicates that the community assembly of fungi and bacteria is a complex and dynamic process, which may be affected by a combination of several factors such as environmental factors, inter-species interactions, stochastic diffusion, and drift, and no single factor can completely dominate the microbial community composition and structure. However, fungal community assembly at the veraison-early stage was dominated by homogeneous dispersal (−2 < βNTI < 2, RC Bray < −0.95), suggesting that the colonization and dispersal of fungi at this time were relatively homogeneous and random, and that the microbial community composition and structure were determined mainly by the dispersal and colonization processes of microbial populations, without being influenced by environmental selection or obvious influence of inter-species competition. It also indicated that the fungal community at the veraison-early stage was relatively stable.

### 3.4. Microbial Co-Occurrence/Co-Antagonistic Interactions

Networks of associations between dominant taxa in microbial ecosystems shape community structure and function with ecological significance. A co-occurrence network was constructed based on correlation coefficients (Spearman’s correlation coefficient r ≥ 0.6, *p* < 0.05) by excluding unidentified fungal and bacterial genera and those with relative abundance below 0.01%. The preliminary prediction of the co-occurrence and exclusion of grape microbiome interactions during the growth season was used to infer the possible “collaboration” or “competition” among the different microbial members. Network nodes indicate microbial genera, and node colors reflect different microbial phyla. Node size indicates its degree of connectivity, the larger the node, the higher its importance for community construction. The edge represents the co-occurrence association between microbial genera, with red color showing a positive correlation and blue color indicating a negative correlation (Supplementary Figures S7 and S8). Overall, the modularity index of all the networks is higher than 0.4, indicating that these networks have a modular structure of well-connected nodes and create a “small-world” topology.

**Figure 6:**
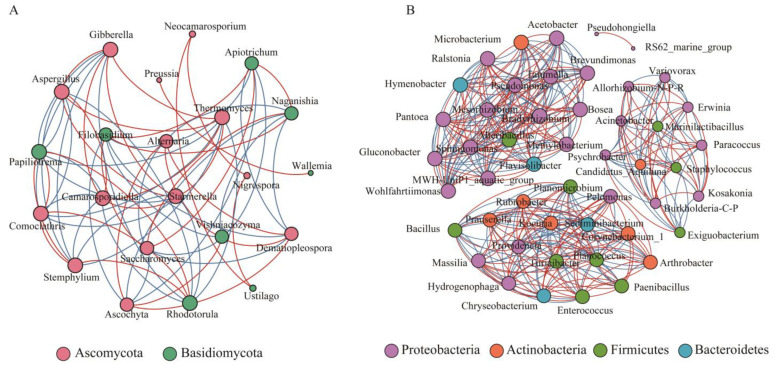
Co-occurrence networks of microbial genera at harvest stage. (**A**) Fungi; (**B**) bacteria. Node size indicates its degree of connectivity, the larger the node, the higher its importance for community construction. The edge represents the co-occurrence association between microbial genera, with red color showing a positive correlation and blue color indicating a negative correlation.

The average degree of the fungal network at the fruit-set stage was found to be 6.875 through the fungal interaction network at each period, and a total of 13 nodes had high connectivity, mainly including *Hannaella*, *Tulostoma*, *Neocamarosporium*, *Stemphylium*, and *Alfaria*, which were mainly positively correlated (87.27%) (Supplementary Table S2). However, Erysiphe, a harmful fungus, also had the highest connectivity, and although its abundance was relatively low, it was able to influence the construction of the fungal network at the fruit-set stage. The fungal network at the veraison-early stage was simpler, with an average degree of 2.615, and the fungal genera with high connectivity were *Gibberella*, *Alternaria*, *Ascochyta*, *Aureobasidium*, and *Papiliotrema*, with *Gibberella* significantly negatively correlated with *Alternaria*, *Ascochyta*, and *Aureobasidium* and significantly positively correlated only with *Papiliotrema*. Furthermore, *Saccharomyces* was positively associated with *Apiotrichum* and *Hanseniaspora*, different from the negative association of *Saccharomyces* with *Hanseniaspora* during the fermentation process. The average degree of the fungal network at the veraison-end stage was 2.333, and the network was still relatively simple. *Alternaria* was significantly positively connected to *Aureobasidium*, both of which were significantly negatively correlated with *Saccharomyces*, and *Gibberella* was significantly positively correlated with *Saccharomyces*. At mid-maturity, the fungal network became progressively more complex, with an average degree of 4.533 and higher connectivity of seven fungal genera of lower relative abundance, mainly *Papiliotrema*, *Sympodiomycopsis*, *Thermomyces*, and *Coprinellus*, some of which are yeasts or yeast-like fungi that do not generally result in significant grape health. During the harvest stage, the average degree of the fungal network was 6.182, at which time the negative relationship pairs between fungal genera increased significantly, and nine genera, including *Gibberella*, *Camarosporidiella*, *Rhodotorula*, *Comoclathris*, *Papiliotrema*, and *Aspergillus*, were more connected (Figure 6A). *Camarosporiosis*, one of the pathogens of grapevine black rot, was found to be positively associated with *Aspergillus*, *Thermomyces*, and *Starmerella*, which should be emphasized and harvested at the right time. *Filobasidium* and *Naganishia* were significantly positively associated, and both were significantly negatively associated with *Alternaria* and *Ascochyta*. In addition, *Gibberella* was positively associated with *Starmerella* and *Aspergillus*, and *Rhodotorula* was negatively associated with *Starmerella* and *Aspergillus*.

The structure of bacterial network was more complex compared to fungi, and the average degree of bacterial network at the fruit-set stage was 24.351, with a total of 32 bacterial genera reaching 31 connecting nodes, mainly including *Sediminibacterium*, *Bradyrhizobium*, *Prauserella*, *Gluconobacter,* and *Alteribacillus*. *Sediminibacterium* was significantly positively correlated with *Bradyrhizobium* and significantly negatively correlated with *Gluconobacter* (Supplementary Table S3). *Ralstonia* and *Gluconobacter* were significantly negatively correlated mainly with several bacterial genera, and *Prauserella* was significantly positively correlated with several bacterial genera. The bacterial network at the veraison-early stage became complex, reaching 143 nodes and 2226 edges with an average degree of 31.133. *Sediminibacterium*, *Ralstonia*, *Pantoea*, *Mesorhizobium*, *Erwinia,* and *Pseudomonas* had the highest number of associated nodes. *Sediminibacterium* and *Ralstonia* were significantly negatively correlated with *Pantoea* and *Lactobacillus*, and *Pantoea* was positively associated with *Erwinia* and *Pseudomonas*. The bacterial network at the veraison-end stage reached 147 nodes with 2158 edges and an average degree of 29.361, of which 39 nodes had the highest connectivity, with the main connected nodes being *Ralstonia*, *Brevundimonas*, *Mesorhizobium*, *Bosea*, *Massilia*, and *Pseudomonas*, among others. Many rare bacterial genera with low relative abundance carried the network construction. *Brevundimonas* was significantly negatively associated with *Pantoea* and *Pseudomonas*. *Bosea* was positively associated with *Pantoea* and negatively associated with *Massilia*. The bacterial network in the middle stage of maturation exhibited a gradual trend towards simplicity with an average degree of 21.375, with *Sediminibacterium*, *Bradyrhizobium*, *Pantoea*, *Brevundimonas*, *Sphingomonas*, and *Allorhizobium*-*Neorhizobium*-*Pararhizobium*-*Rhizobium* being the major connecting nodes. *Bradyrhizobium* was significantly positively correlated with *Pantoea*, and *Sphingomonas* was significantly negatively correlated with *Allorhizobium*-*Neorhizobium*-*Pararhizobium*-*Rhizobium*. The structure of the bacterial network at harvest time was relatively simple, with only 49 nodes and 356 edges, and the average degree was only 14.531. *Pantoea*, *Ralstonia*, *Bradyrhizobium*, *Sphingomonas*, *Brevundimonas*, and *Gluconobacter* were the main bacterial nodes at harvest time (Figure 6B). *Pantoea* was positively associated with *Sphingomonas* and significantly correlated negatively with *Gluconobacter*. *Planomicrobium* was significantly and positively associated with *Planococcus* and both of them were significantly and negatively associated with *Sediminibacterium*. Moreover, *Gluconobacter* had strong positive associations with *Acetobacter* and *Tatumella*, but significant negative associations with *Pseudomonas* and *Methylobacterium*, which are potential biocontrol agents.

**Figure 7:**
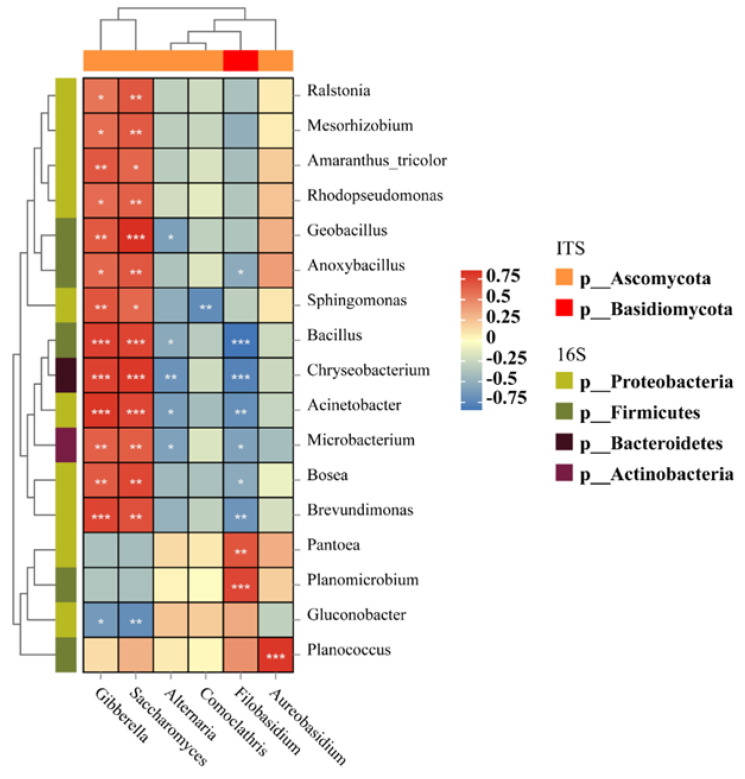
Correlation heat map of dominant fungal and bacterial genera. The microbial genera contained in the relationship pairs with top30 correlations were filtered. The horizontal/vertical axes indicate fungi and bacteria, respectively, and the color of the squares within the plot indicates the correlation strength. The color of the axis labeled species indicates the species annotation at the corresponding phylum level. * *p* < 0.05; ** *p* < 0.01; and *** *p* < 0.001.

In general, the complexity of the fungal network during growing season showed a trend of decreasing and then increasing, and the bacterial network showed a trend of increasing and then significantly decreasing, which is in line with the results of changes in microbial diversity. The correlation analysis between the dominant fungal and bacterial genera during growth revealed that the fungal genera *Gibberella* and *Saccharomyces* were significantly and positively correlated with a variety of bacterial genera, such as *Brevundimonas*, *Bosea*, *Microbacterium*, *Acinetobacter*, *Chryseobacterium*, *Bacillus*, and *Sphingomonas*, but all were significantly and negatively correlated with *Gluconobacter* (Figure 7). *Alternaria* revealed a significant negative association with *Chryseobacterium*. *Filobasidium* expressed a significant positive association with *Pantoea* and *Planomicrobium*, and *Aureobasidium* showed a significant positive association with *Planococcus*. The interactions between microorganisms during the growth period may indicate physiological and metabolic relationships between them that influence survival status and species succession among microbial members.

### 3.5. Correlation of Weather Parameters with Microbial Communities

**Figure 8:**
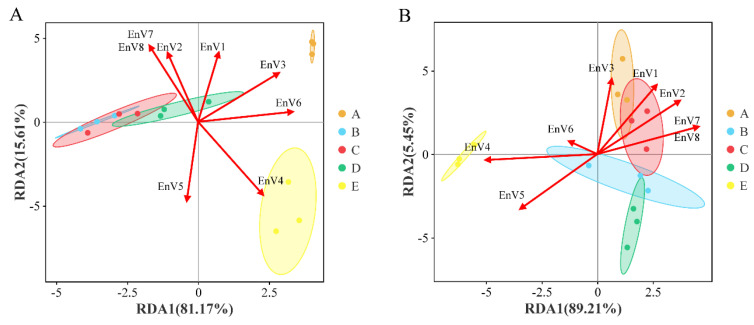
Correlation of weather parameters with microbial communities during the growth phase as indicated by redundancy analysis (RDA). (**A**) Fungi; (**B**) bacteria. Abbreviations: EnV1, mean temperature; EnV2, mean high temperature; EnV3, mean low temperature; EnV4, precipitation; EnV5, relative moisture; EnV6, evaporation; EnV7, solar radiation; and EnV8, sunlight hours.

**Figure 9:**
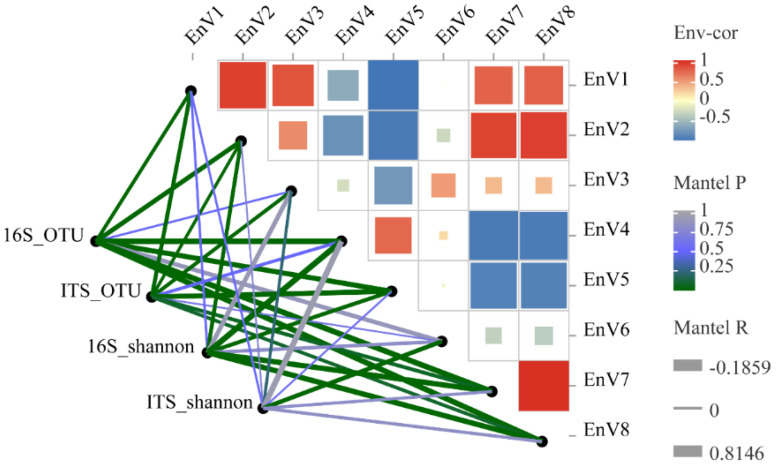
Mantel test analysis of fungal and bacterial community diversity and weather conditions. EnV1, mean temperature (°C); EnV2, mean high temperature (°C); EnV3, mean low temperature (°C); EnV4, precipitation (mm); EnV5, relative moisture (%); EnV6, evaporation (mm); EnV7, solar radiation (J/m2); and EnV8, sunlight hours (h).

The structure and composition of the microbial community change significantly during grape growing stages, and the microbiome succession of different growth and metabolic types is related to the external conditions to which it is exposed. The microclimate of the grapevine plant promotes or antagonizes the colonization of different ecotypes of fungi and bacteria, which may also contribute to the natural state of the grape microbiome. In this study, fungal and bacterial communities of grape epidermis were correlated with weather conditions during each growing stage. RDA combined with the Envfit test for significance indicated that fungal and bacterial communities were significantly correlated with several weather indicators (except for bacterial communities, which were not significantly correlated with evaporation) (Figure 8) (Table 2). Mantel test analysis based on Pearson correlation showed that relative moisture was significantly correlated with the effect of ITS_OTU (r = 0.693, *p* = 0.001), while ITS_Shannon was mainly affected by evaporation (r = 0.6, *p* = 0.005). The highest contribution of precipitation was observed for 16S_OTU (r = 0.797, *p* = 0.001) and 16S_Shannon (r = 0.815, *p* = 0.001), followed by solar radiation and sunlight hours (Figure 9). In addition, the Mantel test showed a lower degree of correlation between fungal communities and weather parameters (r = 0.398, *p* = 0.023), while a higher correlation was found between bacterial genera and weather parameters (r = 0.837, *p* = 0.001) (Supplementary Figure S9). These results reflect the fact that the bacterial community is more susceptible to fluctuations in weather conditions during the growing season, while the fungal community is relatively steady. Overall, the bacterial community was mainly impacted by precipitation and the fungal community was mainly affected by evaporation, as evidenced by variance partition analysis (VPA) (Supplementary Figure S10).

**Figure 10:**
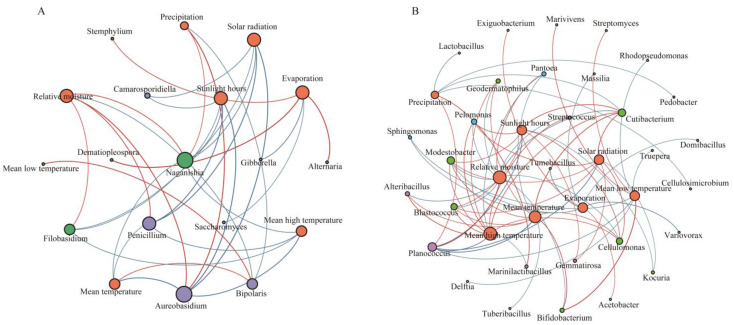
Correlation network between natural microorganisms and weather parameters during growth stages. (**A**) Fungi; (**B**) bacteria. The different colored nodes indicate weather parameters and microbial genera, and the node size indicates the degree of connectivity between them, with red edges indicating a significant positive correlation between the two and blue, a significant negative correlation.

Microbial genera with relative abundance >0.01% were selected for Spearman’s correlation analysis with standardized environmental factors, and correlation coefficients matrices with correlation coefficients |r| > 0.6 and *p* < 0.05 were screened out to construct a statistically robust correlation network (Figure 10). The correlation network between microbial communities and weather factors revealed that 11 fungal and 30 bacterial genera were significantly correlated with weather parameters. The results showed that *Gibberella* and *Saccharomyces* with higher relative abundance were both significantly negatively correlated with evaporation. *Alternaria*, *Dematiopleospora*, and *Stemphylium* were all significantly positively correlated with evaporation. *Aureobasidium*, *Filobasidium*, *Naganishia*, and *Penicillium* were significantly positively correlated mainly with precipitation and relative moisture and significantly negatively correlated with mean high temperature, solar radiation, and sunlight hours. Moreover, relative moisture was significantly negatively correlated with several bacterial genera but positively correlated with *Pantoea* and *Planococcus*. Several bacterial genera such as *Alteribacillus*, *Streptococcus*, *Blastococcus*, *Cellulomonas*, *Cutibacterium*, and *Geodermatophilus* were significantly and positively associated with mean high temperature, mean temperature, and solar radiation. It was also observed that *Lactobacillus* was significantly negatively correlated with precipitation, *Acetobacter* was significantly positively correlated with evaporation, while *Massilia*, *Variovorax*, and *Sphingomonas* were significantly negatively correlated with evaporation.

### 3.6. Prediction of Microbial Community Metabolic Function

**Figure 11:**
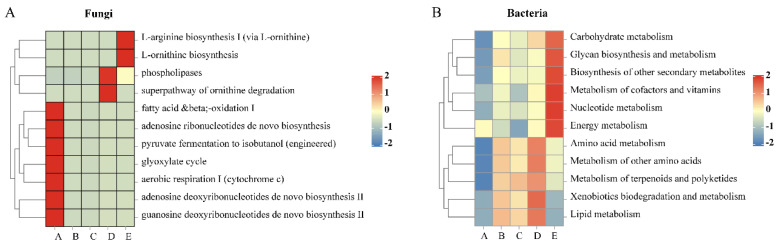
Heat map of the functional abundance of the main metabolic pathways of the microbial community during grape growth: (**A**) fungi and (**B**) bacteria.

Microbial community-enriched metabolic pathways during grapevine fruit growth regulate the synthesis and accumulation of secondary metabolites and biochemical transformations, thus being able to balance the stability of microbial ecosystems under different environmental conditions and developmental stages. Preliminary predictions of metabolic pathways in fungal (based on the MetaCyc pathway database) and bacterial communities (based on the KEGG pathway database) were made by PICRUSt2 (Figure 11). The results indicated that a total of 78 pathways existed in the fungal community, with major functional abundances prominently focused on the fruit-set stage, such as aerobic respiration I (cytochrome c), fatty acid beta-oxidation I, glyoxylate cycle, adenosine deoxyribonucleotides de novo biosynthesis II, adenosine ribonucleotides de novo biosynthesis and guanosine deoxyribonucleotides de novo biosynthesis II, etc., which suggests that the fungal community undergoes active energy metabolism to supply its own growth and energy during the fruit-set stage. Fungal metabolic functions were essentially stable during the veraison stage, with a relative increase in the abundance of L-ornithine biosynthesis and L-arginine biosynthesis I (via L-ornithine) during the harvest.

In contrast to the fungal community, the primary metabolic abundance of the bacterial community was significantly lower at the fruit-set stage. Thereafter, it was basically divided into two categories, one of which was generally a gradual increase in the metabolic function abundance from the veraison-early to the harvest stages, mainly including carbohydrate metabolism, metabolism of cofactors and vitamins, nucleotide metabolism, and energy metabolism. In addition, amino acid metabolism, xenobiotics biodegradation and metabolism, lipid metabolism, and metabolism of terpenoids and polyketides increased significantly in abundance from the veraison-early to mid-maturity stages but decreased significantly at harvest. These results suggest that changes in the metabolic functions of the microbial community may reflect fruit ripening and that microbes are involved in the metabolic transformation of fruit nutrients and may be important in regulating the grape cluster microenvironment, increasing the flavor characteristics and producing antimicrobial substances to inhibit the growth of harmful microbes.

## 4. Discussion

The microbial community of the grape epidermis has been extensively studied, focusing mainly on the effects of different grape varieties and different climatic origins of grapes on the microbial community composition and diversity. However, it has still not been possible to give clear, generalized conclusions on the sequential succession patterns of the grape microbiota and their influence on physiological and metabolic properties at various stages of berry development, since the grape microbiota may be affected by a combination of factors such as vineyard climates, geographic locations, grape varieties, and cultivation practices [29,30,31]. In particular, the microbiome of Ecolly, a white grape variety with disease- and cold-resistant characteristics that is widely grown in northwestern China, remains poorly studied [32]. The main objective of this study was to investigate the dynamic patterns of the grape epidermal microbiome during berry development under an extremely simplified cultivation model for Ecolly grapes adapted to the geographic and climatic characteristics of northwestern China. It will provide references and reflections for the varietal promotion of Ecolly, filling in the evidence of microbial advantages of extremely simplified eco-cultivation and constructing a sustainable system of viticulture in China.

Our results suggest that the grape microbiome structure and diversity are strongly influenced by the developmental stage of the fruit, which is consistent with the research findings of Carmichael et al. [33] and Wei et al. [34]. A distinct separation of fungal and bacterial community structure occurred during the fruit-set stage to the veraison-early stage, indicating a change in grape physiology and biochemistry that may have been selected for epiphytic microorganisms. It is at this time that grape berries are rapidly growing and expanding, with cell division occurring concurrently with cell enlargement, and accumulating nutrients such as glucose, fructose, malic acid, tartaric acid, and phytohormones [35]. Meanwhile, microbial functional metabolism was very active during the fruit-set stage, and fungi were mainly involved in aerobic respiration I (cytochrome c), fatty acid beta-oxidation I, glyoxylate cycle, and guanosine deoxyribonucleotides de novo biosynthesis II, among others, suggesting complex regulation and adaptation of energy production, organic matter catabolism, and nucleic acid synthesis in fungi. From the veraison-early to the mid-maturity stages, along with the gradual ripening of the fruit, the microbial community enters a completely different survival environment where conditions such as the epidermis becoming thinner, the berries expanding again, the sugar content increasing, the acidity decreasing, and the accumulation of aromatic substances lead to a significant increase in the abundance and diversity of the fungal community [13,36]. The abundance of basidiomycetous yeasts such as *Filobasidium* and *Naganishia* was detected to begin to dominate in this process. However, bacterial community diversity tended to decrease from veraison to harvest, probably related to the weather conditions in the vineyard, especially the significant correlation of 16S_OTU and 16S_Shannon of bacteria with temperature, precipitation, solar radiation, and sunlight hours, which is more susceptible to fluctuations in environmental conditions as compared to fungi. In summary, the veraison stage is a critical point in the entire grape growth process where the microbial community is significantly changed. Microbial communities undergo relay-order succession during grape development, and there is potential consistency in the response of fungal and bacterial community structures to the growing stage.

Several environmental and anthropogenic factors can influence the diversity and richness of the grape microbiome, and the common microbial genera found on the grape epidermis are well known [31]. Zhu et al. [37] studied the epiphytic microbiome of Cabernet Sauvignon from budding to ripening and found that *Aspergillus*, *Malassezia*, *Metschnikowia*, and *Udeniomyces* were predominant during the unripe stage, whereas *Erysiphe*, *Cryptococcus*, *Vishniacozyma*, and *Cladosporium* were dominant in the ripe stages. The research results of Liu and Howell [13] also found that the diversity of the fungal community on Pinot Noir showed a gradually increasing trend from the fruit-set to the harvest stages, mainly *Aureobasidium*, *Cladosporium*, *Epicoccum*, *Cryptococcus,* and *Alternaria*. Moreover, it is considered that veraison appears to be a critical stage in which the core community is different from other stages, which is consistent with our findings. In our study, epiphytic microbes were dominated by *Gibberella*, *Alternaria*, *Filobasidium*, *Naganishia*, *Ascochyta*, and *Apiotrichum* as the dominant fungal genera and *Sediminibacterium*, *Ralstonia*, *Pantoea*, *Bradyrhizobium*, *Brevundimonas,* and *Mesorhizobium* as the dominant bacterial genera, which highlights the influence of different regions, grape varieties, and growing stages on natural microbiome. A study by Wei et al. [34] on the microbial diversity and dynamics during the growth of Cabernet Sauvignon at this winery found that the fungi were mainly *Alternaria*, *Jattaea*, *Clavispora*, *Naganishia,* and *Filobasidium* and the bacteria were mainly *Allorhizobium*-*Neorhizobium*-*Parhizobium*-*Rhizobium*, *Brevundimonas*, *Sphingobacterium*, *Acinetobacter,* and *Pseudomonas*. This result that we found *Filobasidium* and *Naganishia* to be widely present in the grape epidermis at harvest time in this zone is consistent with the findings of Wei et al. [34].

Although many studies have been conducted to detect the species composition of grape microbial communities, few studies have concluded a dynamic succession pattern of natural microorganisms during growing stages. While this could be influenced by multiple factors, it is necessary to summarize longitudinally the microbial succession and sequential shifts over time. In our study, several filamentous fungi such as *Alternaria*, *Apiotrichum*, *Ascochyta*, *Comoclathris*, and *Malassezia* were mainly present at the fruit-set stage in relatively even numbers, as well as small abundances of *Filobasidium* and *Hannaella*. The abundance of some specific fungi increased at the veraison stage, in particular *Gibberella* and *Aureobasidium*, and small amounts of *Saccharomyces* and *Erysiphe* were also found. The structural changes in the fungal community during the veraison stage may have a significant impact on grape berry quality and health. At mid-maturity, the fungal population was relatively stable, but the composition changed, with a decrease in *Gibberella* and *Saccharomyces* and an increase in the abundance of *Alternaria*, *Filobasidium*, and *Aureobasidium*. At the harvest stage, mainly basidiomycetous yeasts dominated, including *Filobasidium* and *Naganishia*, while *Gibberella* decreased significantly and *Alternaria* maintained an increasing trend. In addition, small amounts of *Aureobasidium*, *Camarosporidiella*, and *Rhodotorula* were also present, which should be harvested at the right time to prevent fruit infestation. Therefore, the fungal community during the growth of Ecolly grapes was mainly characterized by the transformation of a group of filamentous fungal genera into specific several filamentous fungi through the selective action of the veraison stage and finally into a process dominated by the basidiomycetous yeasts. The number of ascomycete yeast genera is low during the pre-growth period, but an increasing trend is observed during the growth process, including the relatively low abundance of *Candida*, *Starmerella*, *Vishniacozyma*, and *Saccharomyces* detected during the ripening and harvest stages, which may play an important role in the spontaneous fermentation process [38,39]. However, in the study of bacterial communities, Gram-negative bacteria were dominant, mainly including *Sediminibacterium*, *Ralstonia*, *Bradyrhizobium*, *Pantoea*, and *Brevundimonas*. As the fruit ripened, the abundance of *Sediminibacterium* and *Ralstonia* showed a decreasing trend, while the abundance of Gram-positive bacteria, such as *Planomicrobium* and *Planococcus,* relatively increased, indicating that the abundance of Gram-negative bacteria decreases during grape ripening and Gram-positive bacteria abundance tends to increase, which is consistent with the findings of Martins et al. [40]. The assembly process of microbial communities has been studied, and it was revealed that bacterial and fungal communities are influenced by a wide range of factors (growth processes, weather parameters, co-occurrence, etc.) and that no single factor completely dominates the composition and structure of the microbial community.

The current study on the effect of natural microbes on the quality of grape berries focuses mainly on harmful pathogens. In our study, small amounts of *Erysiphe* and *Camarosporidiella*, which are usually the causal agents of powdery mildew and black rot, were detected, but there were no obvious symptoms in the grape berries, which may be due to low abundance that did not reach the disease-causing population size, but which should be taken into account. *Aspergillus* and *Penicillium*, which had similarly low numbers in this study, are opportunistic pathogens that are usually more abundant on the surface of damaged ripe grapes and are capable of secreting mycotoxins, which cause fruit rot [41,42]. *Alternaria* is one of the more common saprophytic molds on the grape epidermis and is not normally attacked by intact fruit, but nutrient penetration and breakage of mature fruit can promote the growth of *Alternaria* hyphae in the pulp, causing grape wilt [43,44]. Along with high-throughput sequencing in recent years, *Gibberella* has begun to be detected regularly on the grape epidermis, but its impact on grape growth remains unclear. In fact, most of the natural microbes are neutral in their impact on grape health and grape-to-wine conversion, but they may play a role in diluting the population of pathogen populations. *Filobasidium*, *Naganishia*, *Rhodotorula*, and *Aureobasidium* are usually prevalent on the surfaces of healthy grapes, where they have a positive effect on grape growth, producing beneficial metabolites such as antioxidants, growth hormones, gibberellins, and a variety of extracellular enzymes, which inhibit the growth of several phytopathogens and protect grapes from damage [45,46,47].

Studies have shown that some bacteria play an important role in inducing systemic resistance in grapevines against invading pathogens by activating the host defense system prior to pathogen attack, thus increasing plant resistance to pathogen aggression. Some strains of the genera *Pseudomonas*, *Pantoea*, *Acinetobacter*, *Burkholderia*, and *Bacillus* found in the grapevine rhizosphere and phyllosphere exhibited resistance against Botrytis cinerea that causes grape gray rot [48]. *Sediminibacterium* is more abundant in grapes and may have a neutral effect on grape health. However, *Ralstonia* is a soil-borne bacterium, and *Ralstonia* solanacearum in particular is capable of causing wilt or brown rot, which should be taken into account even though grapes are not its main host [49,50]. *Bradyrhizobium* can provide nutrients to plants by participating in nitrogen fixation [51]. *Bradyrhizobium* and *Brevundimonas* were found to have possible beneficial effects on nutrient cycling and phytohormone production in algae [52], and *Brevundimonas naejangsanensis* was identified as a potential detoxifier of ochratoxin, able to biodegrade both ochratoxin A and ochratoxin B, making it a microbial resource with important applications in the food sector [53]. Some strains of *Pantoea* have been shown to be effective biocontrol agents for postharvest diseases of sweet potato and orange [54,55], and *Pantoea ananatis* is able to exert an inhibitory effect against the harmful bacterium Botrytis cinerea in grapevine phyllosphere [56].

Overall, the Ecolly grape berries were able to fully ripen and remain healthy under extremely simplified eco-cultivation, and their natural microbiota were dynamically influenced by the growth and developmental stages. Certainly, microbial ecology was found to vary across wine regions, vintages, and grape varieties, but geographic scale was the main factor influencing the microbiome, while varietal and vintage had relatively little effect, with the exception of weather extremes between short vintages [20]. Comparing the results of our work in 2020 in this region on the microbiota during the growing season of Ecolly [57], we found that the fungal community structure in 2021 was very much in accordance with that of 2020, and, in particular, *Filobasidium* and *Naganishia* dominated the harvests in both years. Furthermore, comparing our studies on the microbiome of Ecolly grapes in 2020 and 2021 in another region of China [58], we found that the diversity of the fungal community and the microbial community structure during the growing season showed relatively consistent trends with the present study, but the microbial community composition was not identical, which could be attributed to the effects of different climates, weather conditions, and locations. In this paper, with reference to previous studies, the composition and succession of the main microbes in each growth stage were further analyzed in depth, summarizing the important common and universal features of the diversity and structure of the epidermal microbiome of the Ecolly grapes. It will provide more guidance and be more insightful to understand the microbial ecosystem of grapes by concluding the universal and regular information of the microbiota during the grape growth stages. Continuous observation of Ecolly grapes in this region over several vintages is still needed to better define the evolution of the microbial ecosystems in the Wuhai region and their impact on grape health, which is of great practical importance for scientifically guiding the cultivation practices and highlighting the terroir and sustainability of the grape and wine industry.

## 5. Conclusions

The determination of the microbial diversity and dynamics of the microbial community of Ecolly grapes made it evident that natural microbes are significantly influenced by growth stage. Along with grape ripening, the natural microbial community undergoes a dynamic and continuous succession, with a gradual shift from filamentous fungi to the basidiomycetous yeast, the dominance of Gram-negative bacteria in the early stages, and a tendency towards an increase in Gram-positive bacteria in the ripening stage. Grape microbial interactions, environmental responses, and metabolic functions have important implications for grape growth and health. They provide theoretical support for a comprehensive understanding of the ecological characteristics of the microbial community of Ecolly grapes under an extremely simplified cultivation in the Wuhai region and help to highlight the regional terroir and sustainable, high-quality development of grapes and wines.

## Figures and Tables

**Table 1:** Results of the PERMANOVA test for changes in the fungal and bacterial community structure during the grape growing season.

Microbe	Df	SumsOfSqs	MeanSqs	F Value	R^2^	*p* Value	Significant
Fungi	4	2.2967	0.5742	9.6534	0.7943	0.001	**
Bacteria	4	0.9916	0.2479	18.4511	0.8807	0.001	**

Note: Significance of differences in groups, ** *p* < 0.01.

**Table 2:** The degree of impact and significance of weather parameters with fungal and bacterial communities during the grape growing season.

Factor	Weather Parameter	Fungi		Bacteria	
		Envfit_r2	Envfit_*P*	Envfit_r2	Envfit_*P*
EnV1	Mean temperature	0.6142	0.004	0.8976	0.001
EnV2	Mean high temperature	0.6488	0.005	0.8768	0.001
EnV3	Mean low temperature	0.5872	0.007	0.7654	0.001
EnV4	Precipitation	0.8907	0.002	0.8939	0.001
EnV5	Relative moisture	0.7913	0.001	0.8293	0.001
EnV6	Evaporation	0.421	0.035	0.0732	0.651
EnV7	Solar radiation	0.8568	0.002	0.8353	0.001
EnV8	Sunlight hours	0.8566	0.002	0.8351	0.001

## Data Availability

The original contributions presented in the study are included in the article/supplementary material, further inquiries can be directed to the corresponding author.

## Supplementary Materials

The following supporting information can be downloaded at https://www.mdpi.com/article/10.3390/foods13101580/s1. Supplementary materials have been uploaded with the manuscript, Figure S1: Sampling stages and fruit condition during the growth and development of Ecolly grape berries; Figure S2: Rarefaction curves for fungi (A) and bacteria (B) for each sample during the grape growing stage. The horizontal coordinate indicates the number of sequences sampled, and the vertical coordinate indicates the number of OTUs for the corresponding sequence; Figure S3: Analytical box plots of the evenness of the microbial community during growing stage. The smaller the value, the higher the degree of evenness; Figure S4: Procrustes analysis of microbial communities during growing stage. Dots in the figure indicate the samples, and dots connected by lines indicate the 16S and ITS sequencing results corresponding to one sample; Figure S5: Linear discriminant analysis (LDA) and effect size (LEfSe) to determine bacterial markers for each growth stage. (A) Evolutionary branching maps characterize the biological taxonomic hierarchy of significantly different bacterial taxa, and (B) linear discriminant analysis (LDA) demonstrates the effect size of differential bacterial genera; Figure S6: Microbial community assembly processes based on βNTI and RC Bray during grape growth. (A) Fungi and (B) bacteria; Figure S7: Co-occurrence network of fungal communities at each growing stage. Node size indicates its degree of connectivity. The edge represents the co-occurrence association between microbial genera, with red color showing a positive correlation and blue color indicating a negative correlation. A: Fruit-set; B: veraison-early; C: veraison-end; D: mid-maturity; and E: harvest; Figure S8: Co-occurrence network of bacterial communities at each growing stage. The edge represents the co-occurrence association between microbial genera, with red color showing a positive correlation and blue color indicating a negative correlation. A: Fruit-set; B: veraison-early; C: veraison-end; D: mid-maturity; and E: harvest; Figure S9: Mantel test analysis of microbial communities and weather parameters during grape growth. (A) Fungi and (B) bacteria; Figure S10: Variance partition analysis of fungal and bacterial communities during grape growth. (A) Fungi and (B) bacteria; Table S1: Evaluation of the diversity indices of the epidermal microbiome during the grape growth and development process; Table S2: Overview of topological complementary parameters of fungal networks during grape process; Table S3: Overview of topological complementary parameters of bacterial networks during grape growing process.
