# Supplementary material for: Dynamic Succession of Natural Microbes during the Ecolly Grape Growth under Extremely Simplified Eco-Cultivation
Source: Foods. 2024 May 18;13(10):1580. doi: 10.3390/foods13101580 (PMC11120413; doi:10.3390/foods13101580)
Supplement: Supplementary file 1 [file foods-13-01580-s001.zip › foods-2944333-supplementary.pdf]

Figure S1: Sampling stages and fruit condition during the growth and development of Ecolly grape berries.

Figure S2: Rarefaction curves for fungi (A) and bacteria (B) for each sample during the grape growing stage. The horizontal coordinate indicates the number of sequences sampled and the vertical coordinate indicates the number of OTUs for the corresponding sequence.

Figure S3: Analytical box plots of the evenness of the microbial community during growing stage. The smaller the value, the higher the degree of the evenness.

Figure S4: Procrustes analysis of microbial communities during growing stage. Dots in the figure indicate the samples, and dots connected by lines indicate the 16S and ITS sequencing results corresponding to one sample.

Figure S5: Linear discriminant analysis (LDA) and effect size (LEfSe) to determine bacterial markers for each growth stage. (A): Evolutionary branching maps characterize the biological taxonomic hierarchy of significantly different bacterial taxa; (B): Linear discriminant analysis (LDA) demonstrates the effect size of differential bacterial genera.

Figure S6: Microbial community assembly processes based on βNTI- and RCbray during grape growth. (A) Fungi; (B): Bacteria.

Figure S7: Co-occurrence network of fungal communities at each growing stage. Node size indicates its degree of connectivity; The edge represents the co-occurrence association between microbial genera, with red color showing a positive correlation and blue color indicating a negative correlation. A: Fruit-set; B: Veraison-early; C: Veraison-end; D: Mid-maturity; E: Harvest

Figure S8: Co-occurrence network of bacterial communities at each growing stage. The edge represents the co-occurrence association between microbial genera, with red color showing a positive correlation and blue color indicating a negative correlation. A: Fruit-set; B: Veraison-early; C: Veraison-end; D: Mid-maturity; E: Harvest

Figure S9: Mantel test analysis of microbial communities and weather parameters during grape growth. (A): Fungi; (B): Bacteria

Figure S10: Variance partition analysis of fungal and bacterial communities during grape growth. (A): Fungi; (B): Bacteria. EnV1, mean temperature (°C); EnV2, mean high temperature (°C); EnV3, mean low temperature (°C); EnV4, precipitation (mm); EnV5, relative moisture (%); EnV6, evaporation (mm); EnV7, solar radiation (J/m2) and EnV8, sunlight hours (h).

Figure S1

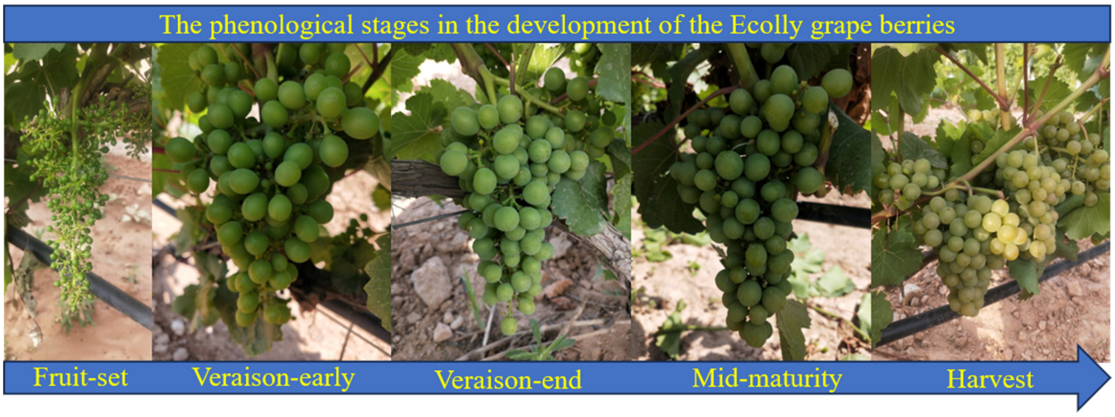

Figure S2

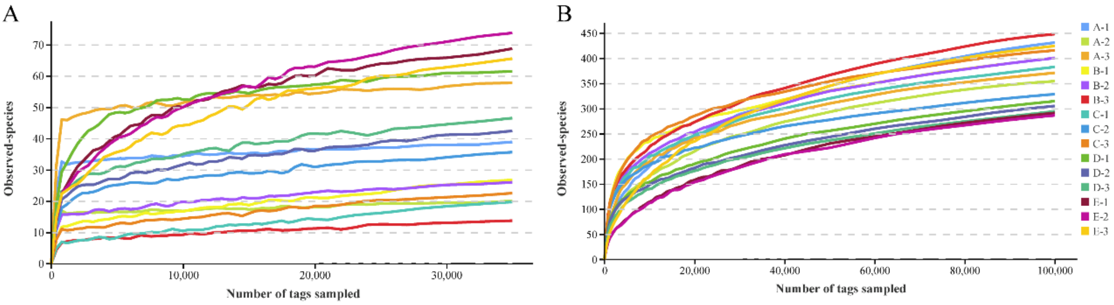

Figure S3

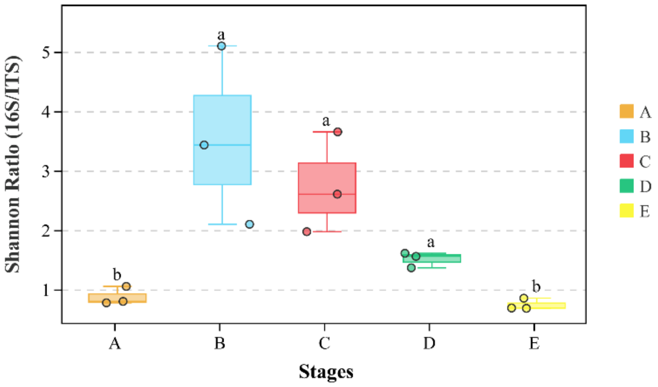

Figure S4

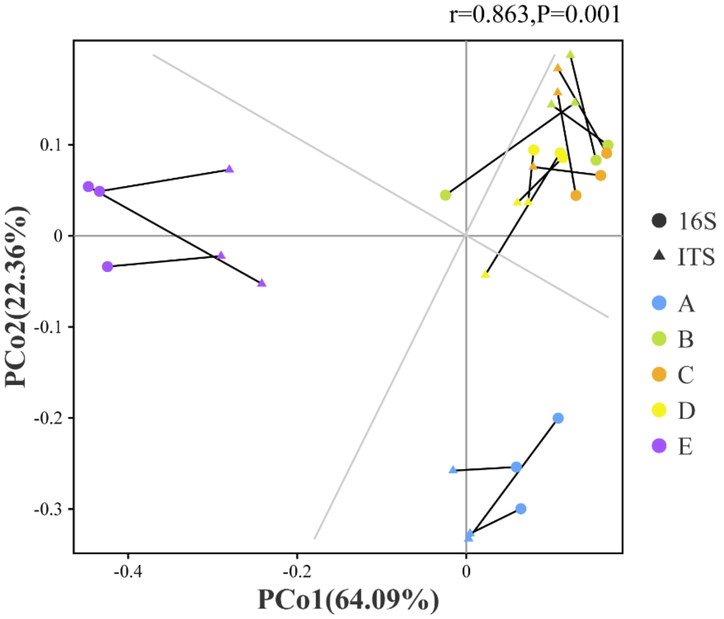

Figure S5

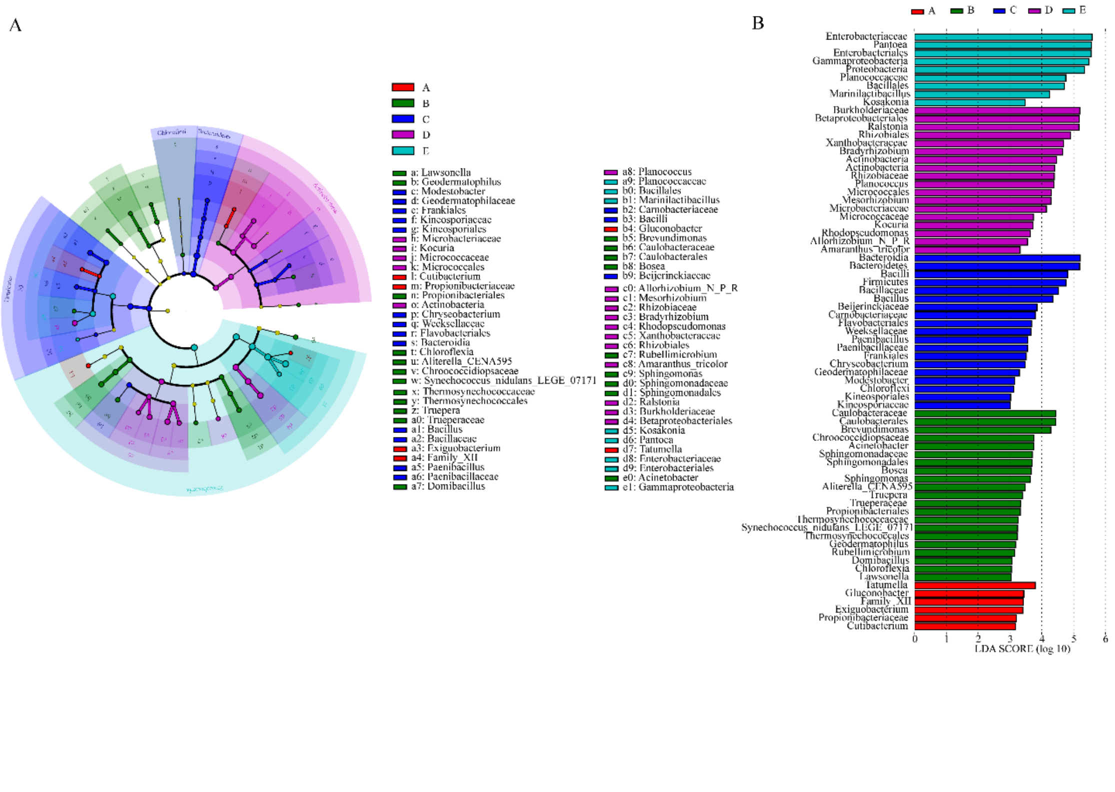

Figure S6

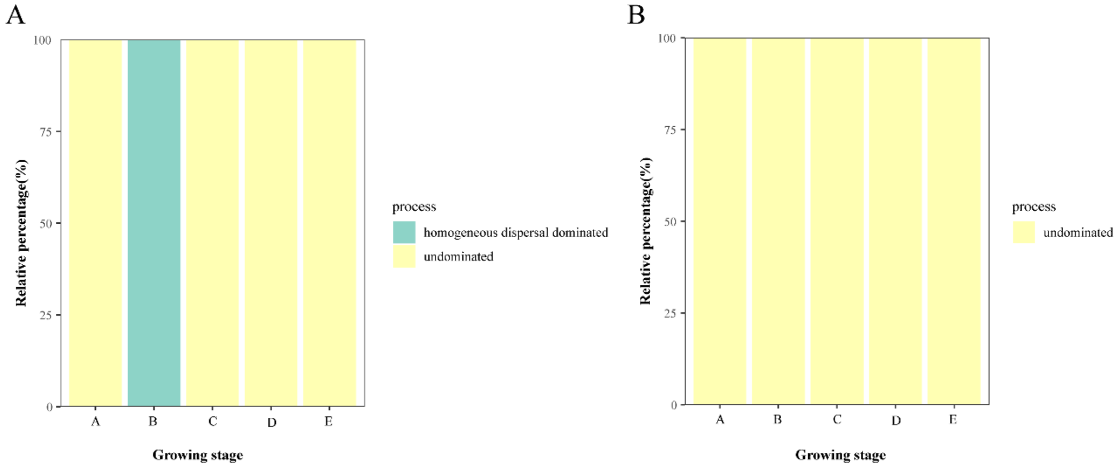
A
B
100
75
50
25
0
Relative percentage(%)
A
B
C
D
E
Growing stage
process
homogeneous dispersal dominated
undominated
process
undominated

.

Figure S7

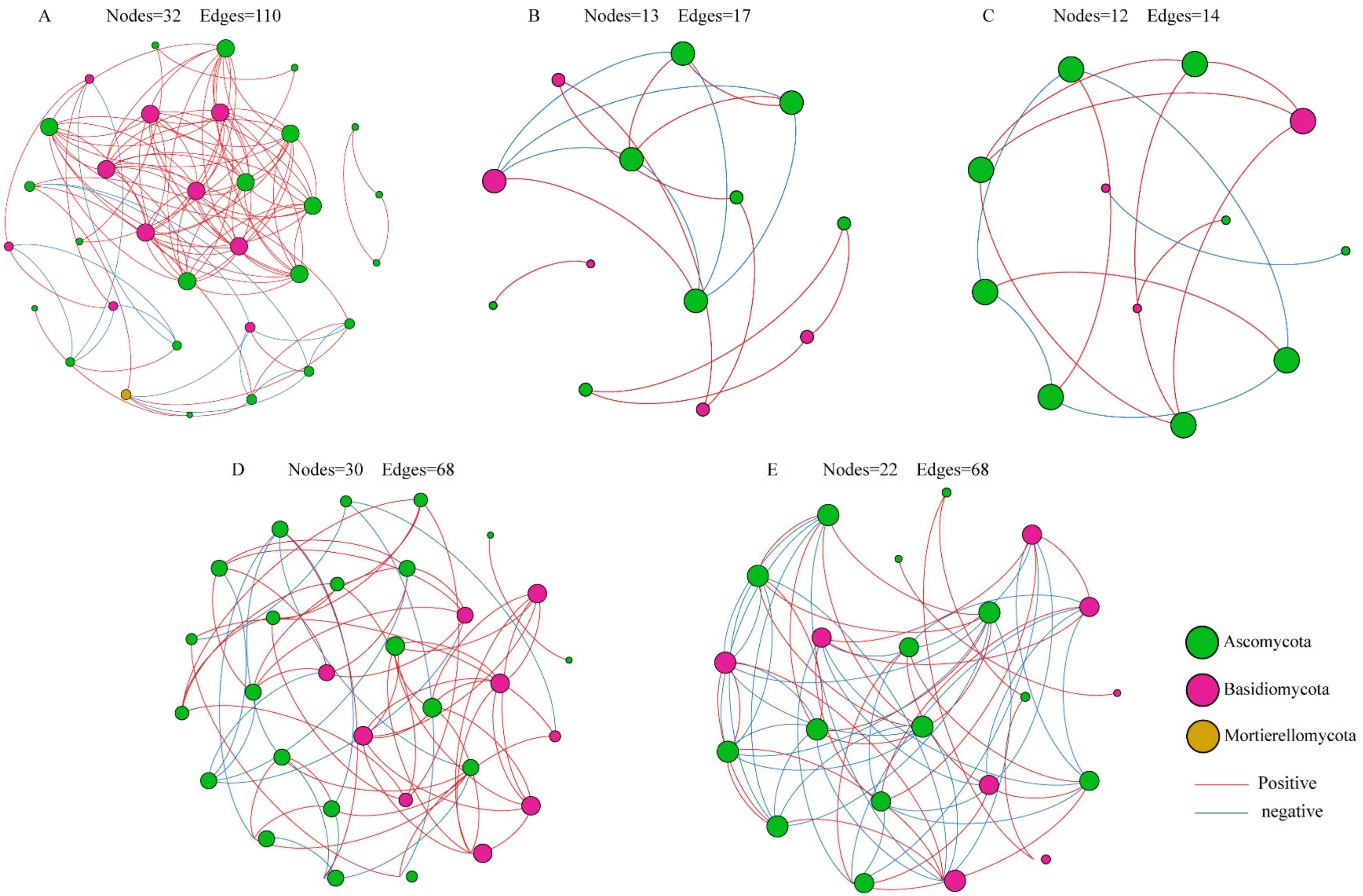
A Nodes=32 Edges=110
B Nodes=13 Edges=17
C Nodes=12 Edges=14
D Nodes=30 Edges=68
E Nodes=22 Edges=68
Ascomycota
Basidiomycota
Mortierellomycota
Positive
negative

Figure S8

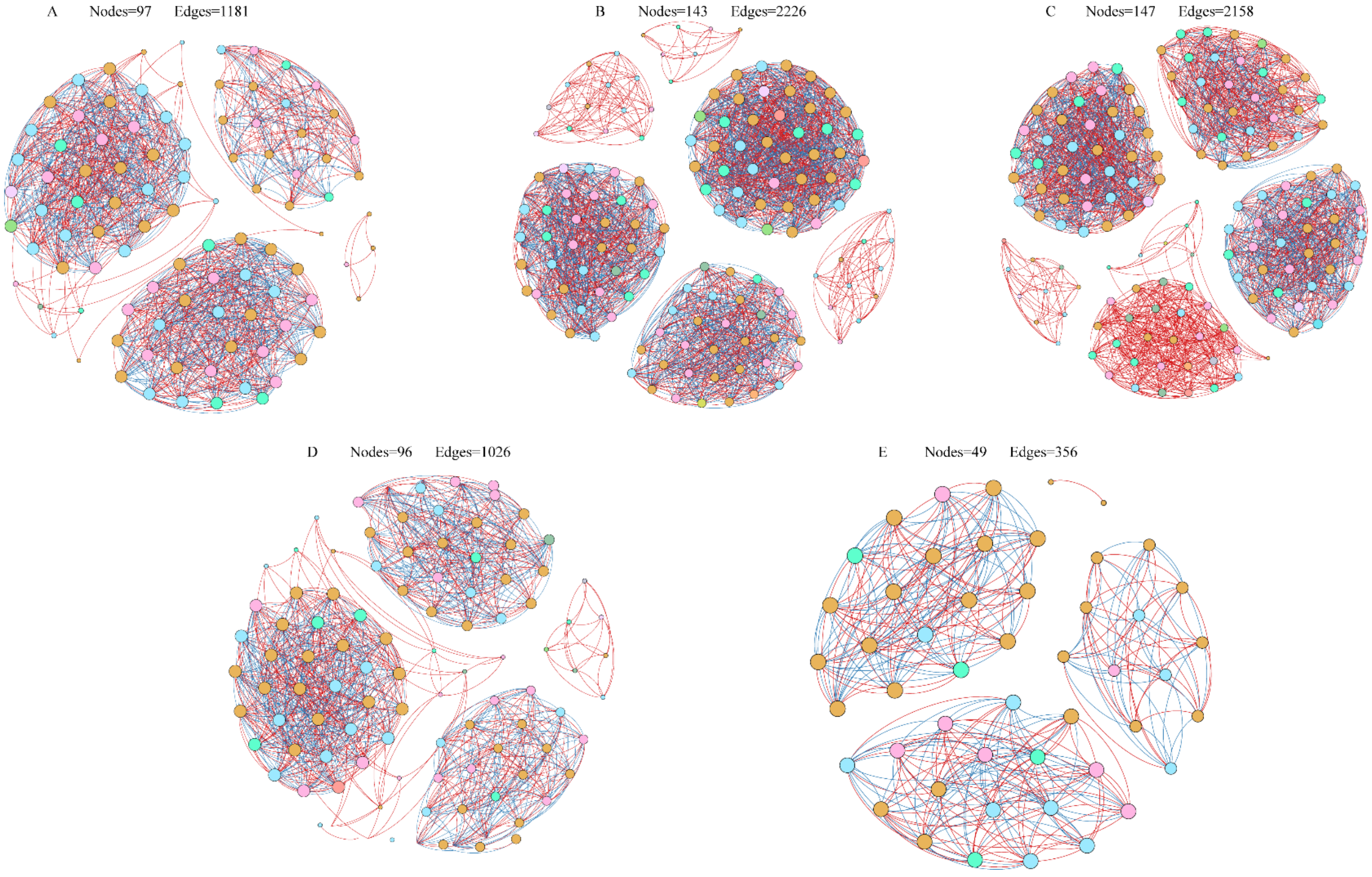

A Nodes=97 Edges=1181
B Nodes=143 Edges=2226
C Nodes=147 Edges=2158
D Nodes=96 Edges=1026
E Nodes=49 Edges=356

Figure S9

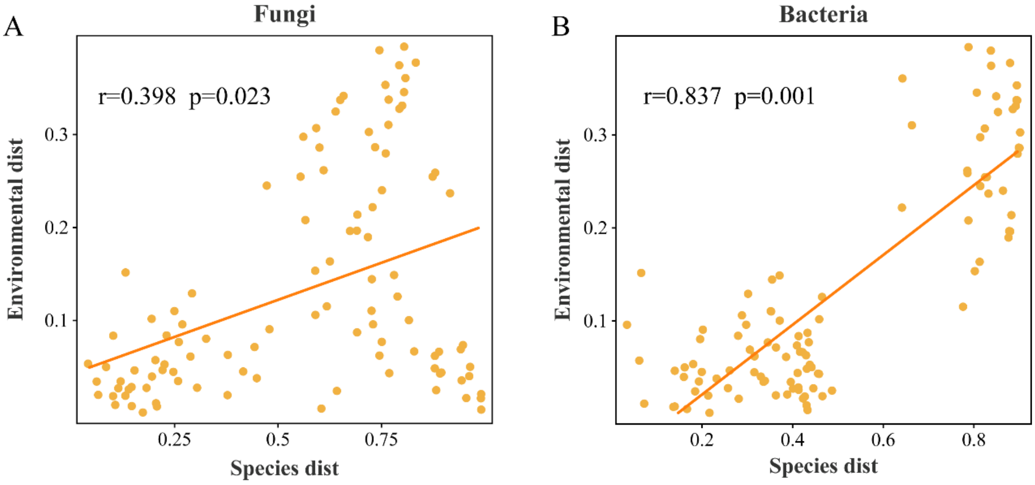

Figure S10

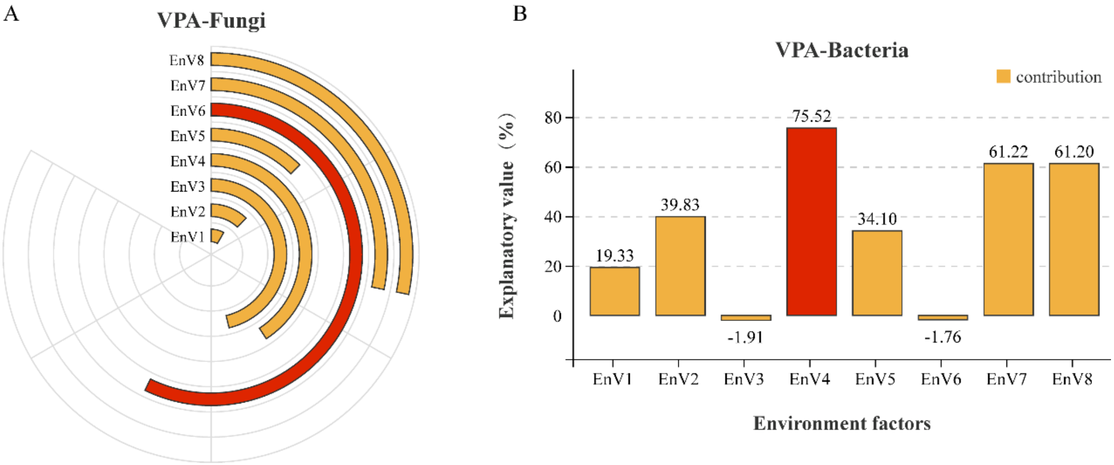

Table S1: Evaluation of the diversity indices of the epidermal microbiome during the grape growth and development process

Microbes	Index	Fruit-set	Veraison-early	Veraison-end	Mid-maturity	Harvest	*F value*	*P value*
Fungi	Chao1	52.67±10.61b	45.58±1.12b	45.29±5.15b	80.58±12.96ab	97.79±5.56a	8.24	**0.003**
	ACE	59.38±10.64b	54.05±9.37b	54.18±6.47b	78.98±8.22ab	98.14±4.92a	5.66	**0.012**
	Shannon	3.62±0.28a	1.26±0.26d	1.51±0.25cd	2.44±0.16bc	2.55±0.02b	18.55	**＜0.001**
	Simpson	0.86±0.02a	0.38±0.06b	0.46±0.07b	0.69±0.04a	0.76±0.01a	21.71	**＜0.001**
	Observed species	45.00±9.24b	29.67±3.38b	32.67±5.24b	54.67±8.17b	87.00±4.62a	12.54	**0.001**
Bacteria	Chao1	456.04±35.04a	545.94±28.29a	448.88±31.78a	403.85±6.39a	406.19±44.98a	3.25	0.059
	ACE	468.99±35.59a	554.94±26.50a	472.48±33.92a	416.07±2.48a	417.66±40.82a	3.34	0.056
	Shannon	3.13±0.07b	4.01±0.23a	3.89±0.24ab	3.66±0.06ab	1.90±0.16c	25.97	**＜0.001**
	Simpson	0.76±0.02a	0.84±0.03a	0.82±0.04a	0.83±0.00a	0.46±0.04b	34.07	**＜0.001**
	Observed species	393.33±23.95a	430.67±17.68a	385.67±28.50a	314.33±6.17a	341.33±44.86a	2.78	0.086

Note: Data are represented as mean ± standard deviation (n = 3) and different lowercase letters after each row of data indicate significant difference. (Tukey's HSD, $p < 0.05$).

Table S2: Overview of topological complementary parameters of fungal networks during grape process

Growing stage	Network parameters	Value
Fruit-set	Number of nodes	32
	Number of edges	110
	Number of positive correlations	96(87.27%)
	Number of negative correlations	14(12.73%)
	Average degree	6.875
	Density	0.222
	Modularity	0.469
Veraison-early	Number of nodes	13
	Number of edges	17
	Number of positive correlations	11(64.71%)
	Number of negative correlations	6(35.29%)
	Average degree	2.615
	Density	0.218
	Modularity	0.588
Veraison-end	Number of nodes	12
	Number of edges	14
	Number of positive correlations	9(64.29%)
	Number of negative correlations	5(35.71%)
	Average degree	2.333
	Density	0.212
	Modularity	0.622
Mid-maturity	Number of nodes	30
	Number of edges	68
	Number of positive correlations	52(76.47%)
	Number of negative correlations	16(23.53%)
	Average degree	4.533
	Density	0.156
	Modularity	0.778
Harvest	Number of nodes	22
	Number of edges	68
	Number of positive correlations	32(47.06%)
	Number of negative correlations	36(52.94%)
	Average degree	6.182
	Density	0.294
	Modularity	0.548

Table S3: Overview of topological complementary parameters of bacterial networks during grape growing process

Growing stage	Network parameters	Value
	Number of nodes	97
	Number of edges	1181
	Number of positive correlations	637(53.94%)
Fruit-set	Number of negative correlations	544(46.06%)
	Average degree	24.351
	Density	0.254
	Modularity	0.642
	Number of nodes	143
	Number of edges	2226
	Number of positive correlations	1191(53.50%)
Veraison-early	Number of negative correlations	1035(46.50%)
	Average degree	31.133
	Density	0.219
	Modularity	0.688
	Number of nodes	147
	Number of edges	2158
	Number of positive correlations	1351(62.60%)
Veraison-end	Number of negative correlations	807(37.40%)
	Average degree	29.361
	Density	0.201
	Modularity	0.745
	Number of nodes	96
	Number of edges	1026
	Number of positive correlations	526(51.27%)
Mid-maturity	Number of negative correlations	500(48.73%)
	Average degree	21.375
	Density	0.225
	Modularity	0.666
	Number of nodes	49
	Number of edges	356
	Number of positive correlations	178(50%)
Harvest	Number of negative correlations	178(50%)
	Average degree	14.531
	Density	0.303
	Modularity	0.635
